# High-intensity ethanol binge drinking accentuates bone damage in induced apical periodontitis in rats

**DOI:** 10.1016/j.heliyon.2024.e40163

**Published:** 2024-11-06

**Authors:** José Mário Matos-Sousa, Deiweson Souza-Monteiro, Vinicius Ruan Neves dos Santos, Maria Karolina Martins Ferreira, Deborah Ribeiro Frazão, Victória Santos Chemelo, Leonardo de Oliveira Bittencourt, João Daniel Mendonça de Moura, Cristiane do Socorro Ferraz Maia, Fabrício Mezzomo Collares, Luanna de Melo Pereira Fernandes, Rafael Rodrigues Lima

**Affiliations:** a Laboratory of Functional and Structural Biology, Institute of Biological Sciences, Federal University of Pará, Belém, Pará, Brazil; b Laboratory of Inflammation and Behavioral Pharmacology, Institute of Health Sciences, Federal University of Pará, Belém, Pará, Brazil; c Laboratory of Dental Materials, School of Dentistry, Federal University of Rio Grande do Sul, Porto Alegre, Rio Grande do Sul, Brazil; d Laboratory of Neuropharmacology and Behavior, Center for Biological |Health Sciences, State University of Pará, Belém, Pará, Brazil

**Keywords:** Apical periodontitis, Binge drinking, High-intensity drinking, Alveolar bone loss

## Abstract

This study aimed to evaluate the effects of excessive and episodic consumption of ethanol (EtOH, a high-intensity drinking manner) on induced apical periodontitis in rats. Thirty-two animals were divided into the following four groups: control, EtOH, apical periodontitis, and EtOH + apical periodontitis. Ethanol exposure (3 g/kg 20 % w/v EtOH) was performed by orogastric gavage for 3 consecutive days, followed by 4 days of withdrawal for 4 weeks. Lesions were induced by exposing the dental pulp of the lower first molar and by the absence of any treatment/curative for 28 days. Finally, the animals were euthanized, and mandibles were collected. The mandible was divided medially, with one hemimandible being used for micro-computed tomography analysis of the volume of the periapical lesion and bone quality parameters, such as bone volume and trabecular bone assessments; the other hemimandible was used for histological analysis, with a descriptive histopathological analysis of the tissue and the pattern of bone loss presented, as well as an assessment of the collagen content present. The data were subjected to statistical analysis (one-way analysis of variance with Tukey's post-hoc test). Our results showed that the EtOH + apical periodontitis group had a larger volume of periapical lesions than animals that were not exposed to ethanol. Additionally, bone quality parameters showed a reduction in bone volume and thickening of the trabeculae, associated with increased tissue destruction and reduced collagen content in the remnant region of the alveolar bone. These results suggest that exposure to EtOH in a pattern of excessive alcohol consumption is an aggravating factor in apical periodontitis and, consequently, in its progression, the quality and quantity of the alveolar bone remaining in the region of the periapical lesion are the modulating aspects.

## 1 Introduction

Apical periodontitis (AP) is an oral disease resulting from a microbial infection of the root canal system and the host's immune response, in which the disease begins as a local inflammation in the pulp and turns into a larger histopathological lesion, characterized by the destruction of periapical tissues. AP is a highly prevalent disease, affecting at least one tooth in 52 % of the adult global population [[1], [2], [3]]. These periapical lesions are caused by microorganisms originating from inside the root canal and present themselves as barriers that restrict and prevent the spread of these microorganisms to the surrounding tissues. As a defense mechanism, bone resorption occurs, followed by replacement with granulomatous tissue and polymorphonuclear leukocytes [[4], [5], [6], [7]]. Thus, in general, AP is asymptomatic, and the presence of the disease is often unknown, since its diagnosis, in these cases, is based on imaging tests, especially radiography [6], generating risk if it is not diagnosed and treated, leading to pain and even loss of the dental element [8], thus compromising the quality of life of the individual [9]. Moreover, owing to infection, the correlation between AP and systemic health has been observed and studied, and AP can be considered a source of systemic inflammation according to more recent studies [[10], [11], [12], [13]].

Although bacterial infection is the primary cause of the disease [14,15], certain habits of the host, such as smoking and alcohol consumption can influence the development of the disease [16]. As measured by the World Health Organization [17] (WHO) (2019), approximately 2.3 billion individuals are currently alcohol drinkers. The alcohol consumption occurs in more than half of the population in two continents of the world: the Americas, Europe, and the region of Western Pacific. This consumption has been an existing concern since 2010 when the WHO adopted its main theme to pay attention to the harm of ethanol excessive consumption and seek strategies to reduce its impact on health. It is well documented that the harmful effects of alcohol intake depend on multiple factors, which the pattern of consumption plays a fundamental role, conferring hazardous effects, especially in heavy episodic or heavy continuous alcohol intake drinkers [18]. The moderate consumption was defined as up to one and two drinks per day for women and men, respectively [19]. Binge drinking pattern occur in the blood alcohol concentration (BAC) levels of 0.08 % or 0.08 g/dL and above, which the BAC levels are a result of 4 or more drinks for women and 5 or more drinks for men during about 2 h of consumption for adults [20,21]. A recent study showed that this type of consumption increased after social isolation measures were implemented during the COVID-19 pandemic [22]. The heaviest designation consists of heavy and high-intensity (extreme) drinking. Heavy alcohol use comprehends the consumption of 15 binge drinking episodes per week for men and at least 8 binge drinking engagements for women [23]. The Substance Abuse and Mental Health Services Administration (SAMHSA) establishes heavy drinking as 5+ binge drinking episodes in the past month, in a more restrictive drinking [24]. In 2024, the NIAAA recognized the definition of the high-intensity binge-drinking, a subcategory of the binge-type exposure [23]. Extreme drinking is a consumption behavior that reaches high peak BAC (at least double levels of binge drinking standard), as a result of the 10+ drinks for men, and 8+ drinks engagement for women, which brings the worse health risks. Evidence has shown that excessive alcohol consumption can damage mineralized tissues [25,26]. This is directly proportional to the dose consumed, as prolonged and excessive consumption, that can lead to deleterious effects such as decreased bone mass, bone mineral density, and inadequate bone repair [27].

We have demonstrated that heavy continuous alcohol consumption elicits alveolar bone loss and morphometric and histological changes in salivary glands in adolescent female rats [28,29]. Some studies have evaluated the bone damage caused by AP associated with chronic alcohol intake at concentrations of 20–25 %, resulting in exacerbation of the inflammatory response and osteoclast activity, as well as a reduction in bone density [16,[30], [31], [32]].

We also investigated the intermittent extreme binge drinking pattern (3 binge drinking episodes per week; following 4-cycles; BAC: 297.3 ± 34.49 [33]) during adolescence on the alveolar bone of rats. We reported that extreme alcohol binge-drinking pattern can influence the alveolar bone quality in a healthy bone [34,35] and in experimentally induced periodontitis [36].

There are important particularities between the two models of oral disease. Periodontitis is characterized by inflammation of the tissues that support the teeth, caused by the long-lasting accumulation of dental biofilm below the gums, leading to gradual vertical bone loss [36]. AP is an infectious/inflammatory process originating from damage to the dental pulp, which spreads to the tissues surrounding the apex of the tooth, resulting in progressive regional bone loss, which, if left untreated, can result in tooth loss, affecting the individual not only from a dental point of view, but also from a psychosocial perspective [1]. Endodontic disease can be treated in various ways [1,6], but studies show that in cases of more acute disease resulting in greater bone loss, endodontic treatments may be less effective and extraction may be indicated [1]. Considering this possibility, there is an urgent need to identify the possible aggravating factors of the developing disease.

In this context, there is a lack in literature concerning the development and impact of binge-type exposure on AP and the consequent damage to the alveolar bone. We hypothesize that 4-cycles of 3 episodes of high-intensity drinking increases alveolar bone damage in AP disease in adulthood; the null hypothesis being that exposure to ethanol in the form of high-intensity drinking was not capable of maximizing alveolar bone loss in apical periodontitis.

## 2 Materials and methods

### 2.1 Experimental animals

**Fig. 1:**
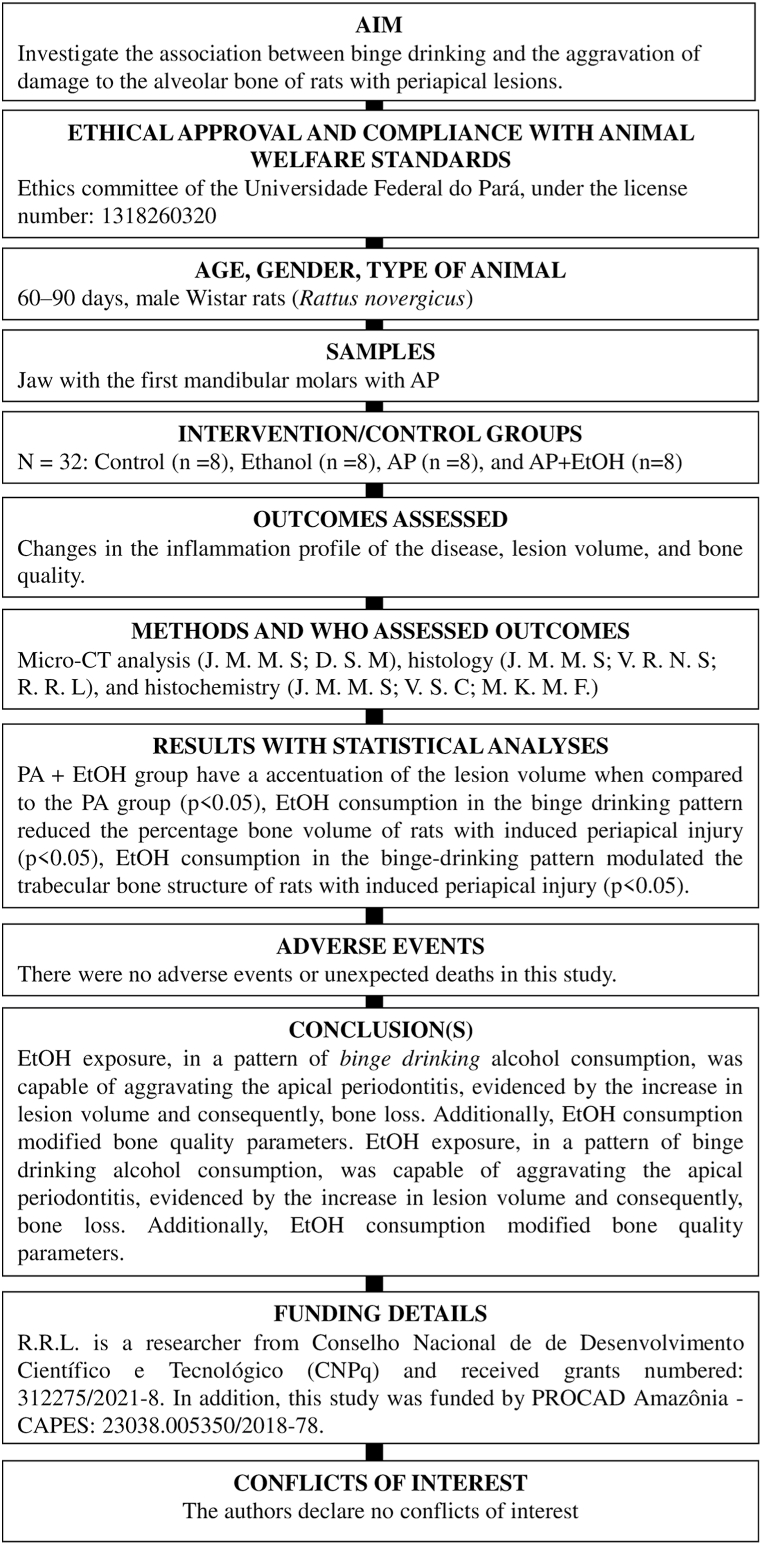
PRIASE guideline flowchart.

The study was approved by the Ethics Committee of the Federal University of Pará (UFPA, Belém-PA, Brazil) under license number CEUA-UFPA: 1318260320 and followed the recommendations of the guideline ARRIVE 2.0 [37]. In addition, this study followed the standardized Preferred Reporting Items for Animal Studies in Endodontology (PRIASE) 2021 guidelines [38], as illustrated in Fig. 1. We used 32 male rats (*Rattus novergicus*) of the Wistar strain, weighing 200–300 g, aged between 60 and 90 days, which were placed in polypropylene cages, with four animals in each cage; lined with shavings and receiving filtered water ad libitum in a controlled manner, staying under light conditions with a light/dark cycle of 12 h and controlled temperature of 25 ± 1 °C.

After acclimatization, the animals were randomly divided into four groups to perform the surgery for lesion induction and the beginning of ethanol (EtOH) administration. The groups (n = 8 animals/group) were divided as follows: Control group; EtOH Group; AP-induced Group; AP-induced, exposed to EtOH Group. The number of animals per group was determined using G∗Power software (Statistical Power Analyses 3.1.9.4) based on the effect size reported by Ref. [36]. An effect size of f = 1.107, a Type I error rate of α = 0.05, and a power of 0.95 were set. These parameters resulted in a total sample size of 20 animals, with 5 animals per group, ensuring a power of 0.968. To keep the study's statistical power intact, we increased the sample size to 8 animals per group to account for potential attrition during the experiment.

### 2.2 Lesion induction

**Fig. 2:**
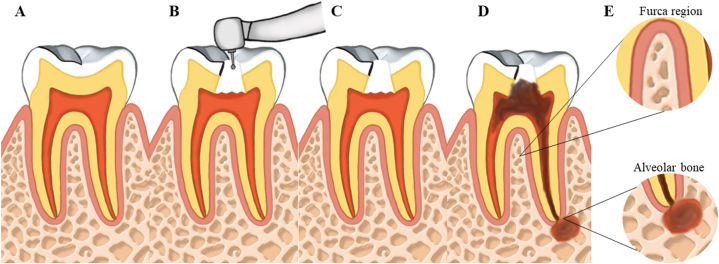
Illustration of the stages of induction of the periapical lesion. A: the tooth is still healthy, B: the access to the pulp chamber with the aid of low rotation, C: the rupture of the pulp tissue, D: the development of the periapical lesion, and E: the analyzed areas of furcation and periapical alveolar bone.

**Fig. 3:**
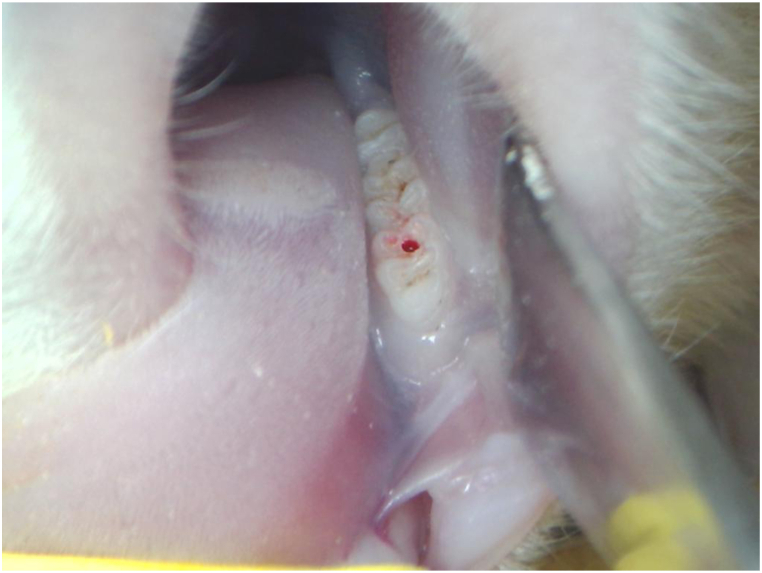
Rat lower first molar after periapical lesion induction procedure.

The animals in the AP and AP + EtOH groups (n = 16) underwent surgery to induce experimental AP on day 0 of the experiment, the day after acclimatization and division of the groups. The animals were anesthetized intraperitoneally with 2 % xylazine hydrochloride (2 mg/mL) and 10 % ketamine hydrochloride (10 mg/mL) at doses of 8 mg/kg (xylazine) and 90 mg/kg (ketamine). The pulps of the left and right first molars were exposed using a #1/4 carbide bur for low rotation coupled to an X-Smart Plus motor (Dentsply Maillefer, Ballaigues, Switzerland). Pulp exposure was confirmed by observing pulp bleeding. The exposed teeth were left open to the oral environment to induce apical lesion formation following the exposure model described in the literature [30,39]. Fig. 2 illustrates the steps of the induction process, and Fig. 3 shows the immediate results after the induction procedure.

### 2.3 High-intensity drinking ethanol protocol

EtOH and AP + EtOH groups were exposed to EtOH at a concentration of 20 % w/v, 3 g/kg/day via orogastric gavage, 5 days after induction of the AP lesion (day 5 of experimentation). Ethanol high-intensity drinking pattern occurred in 4 cycles, with 3 days on and 4 days off per week of EtOH administration, for a total of 28 experimental days. Our group has validated this protocol in several studies [34,36,[40], [41], [42]].

### 2.4 Sample collection and preparation

#### 2.4.1 Perfusion

After completing four cycles of high-intensity drinking, the animals were intraperitoneally anesthetized with 8 mg/kg of xylazine and 90 mg/kg of ketamine. The animals were perfused through the left ventricle of the heart with 0.9 % saline solution heparinized, followed by 4 % formaldehyde.

#### 2.4.2 Collecting the jaws

After perfusion, the mandibles were removed, divided into right and left sides, and prepared by removing adjacent tissues., and fixed in 4 % formaldehyde. The left hemimandible was used to verify the size of the lesion using microtomographic analysis.

#### 2.4.3 Processing and preparation of samples for histopathological analysis

After collection, the right hemimandible was fixed in 4 % formaldehyde for 24 h. Then, they were washed for 4 h in running water and demineralized in 10 % EDTA for 120 days, with a weekly change of liquid, and finally, after this period, washed for 24 h in running water. The specimens were then dehydrated in alcohol (increasing concentrations of 70 %, 80 %, 90 %, absolute I, and absolute II), cleared with xylol, and embedded in paraffin. After processing, the materials were dissected using a Leica RM 2045 microtome (Leica Microsystems, Nussloch - Germany) with a thickness of 6 μm and placed on individual histological slides (Star-frost ®).

### 2.5 Histopathological analysis

For histopathological analysis, the sections were stained with hematoxylin and eosin (HE) and observed under a light microscope. For this analysis, images were obtained using a color digital camera (Cyber-Shot DSC W-230, 4× optical zoom, Sony, Tokyo, Japan) coupled to a microscope (Leica QWin Pl–s-Leica Microsystems, Nussloch, Germany; at 40× magnification) to characterize the bone preservation in the different groups. A trained evaluator underwent an intra-calibration process, performing three repeated analyses on the same sample, focusing on bone preservation and the tooth's apex characteristics. After achieving a satisfactory level of agreement (Kappa ≥0.81), the selection process started.

### 2.6 Histochemical analysis of collagen content

To perform the collagen content of the remaining alveolar bone tissue, the picrosirius red technique was used, for which three randomly selected microscopic sections were utilized for each sample (three sections per animal) for quantitative histomorphometric analysis. These sections were examined under a polarized light microscope at 40× magnification to evaluate collagen content in the remaining alveolar bone. To determine the total collagen area for each sample, ImageJ software was used, and the collagen content was calculated by averaging the threshold percentages from three fields/sections. The result was then measured and expressed in μm^2^.

### 2.7 Computerized microtomography analysis (MicroCT)

The samples were evaluated using X-ray MicroCT (MicroCT.SMX-90 CT; Shimadzu Corp., Kyoto, Japan). Each undecalcified hemimandible was positioned on a rotating base, and images of the sample were taken at 360° rotation with 70 kV intensity and 100 mA. The images were then reconstructed using inspeXio SMX90CT software (Shimadzu Corp., Kyoto, Japan), with 14 μm voxel size, into images with 1024 × 1024-pixel resolution and 14 μm thickness, resulting in 541 images per sample, which were converted to Digital Imaging and Communications in Medicine format.

The CTAn software (V1.15.4.0; Bruker, Kontich, Belgium) was used to reconstruct the alveolar bone area for data analysis. Based on this reconstruction, the positioning of the hemimandibles was standardized such that the periodontal ligament space was highlighted in the section. Thus, following the procedure described by the literature [39], the reconstructed volume of interest (VOI) including the ligament space and area of bone destruction around the roots was defined. Calibration and blinding of the examiner manually delimited the volume of the destroyed area of the mandibular first molar, starting at the mesial root and ending at the distal root. For each evaluated sample, the VOI started with the first coronal section of the mesial root, which was surrounded by the bone crest, proceeded to the distal region, and ended when the mandibular second molar became apparent.

In addition to the evaluation of lesion volume using the CTAn software (V1.15.4.0; Bruker, Kontich, Belgium), analyses were performed to evaluate the quality of the alveolar bone using a group of 220 images of the bone region around the first lower molar. As a region of interest, the alveolar bone around the roots of the molars was defined. For the analysis, the examiner manually delimited the bone in each coronal plane, starting at the proximal point of the mesial root and continuing to the distal point of the distal root. In this analysis, a threshold (31–71) for each image was used to differentiate the cortical bone, trabecular bone, and bone marrow. Quality was assessed using the following parameters: remaining bone volume (VB), percentage of bone volume (VB/TV), trabecular thickness (Tb.Th), number of trabeculae per millimeter (Tb.N), and trabecular spacing (Tb.Sp), which were observed over the remaining bone that was not affected by the lesion.

### 2.8 Statistical analysis

**Fig. 4:**
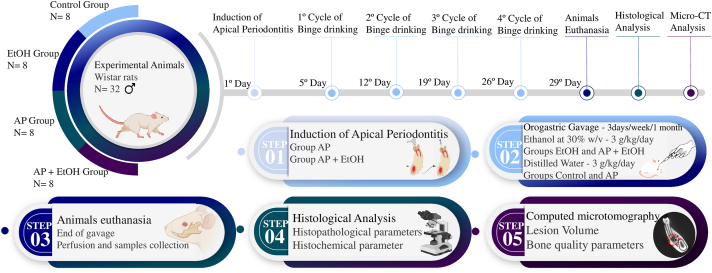
Methodological figure, divided into the chronological line of the experimental days and steps. 1, induction of apical periodontitis (AP) lesion of the AP and AP + EtOH groups; 2, gavage protocol for ethanol exposure; 3, euthanasia and perfusion of the animals, with collection of the mandibles; 4, descriptive histological analysis and histochemical parameter of Picrosirius red; and 5, computed microtomography analysis.

The data from the four experimental groups were tabulated and passed through a normality test using the Shapiro-Wilk method. For parametric results, a one-way analysis of variance (ANOVA) with Tukey's post-test was performed, with a significance value of P < 0.05. All tests were performed in GraphPad Prism 8.0 software and expressed as the mean and standard error of the mean. The methodological steps of this study are illustrated in Fig. 4.

## 3 Results

### 3.1 EtOH consumption in the high-intensity drinking pattern accentuated the volume of the induced periapical lesion in rats

**Fig. 5:**
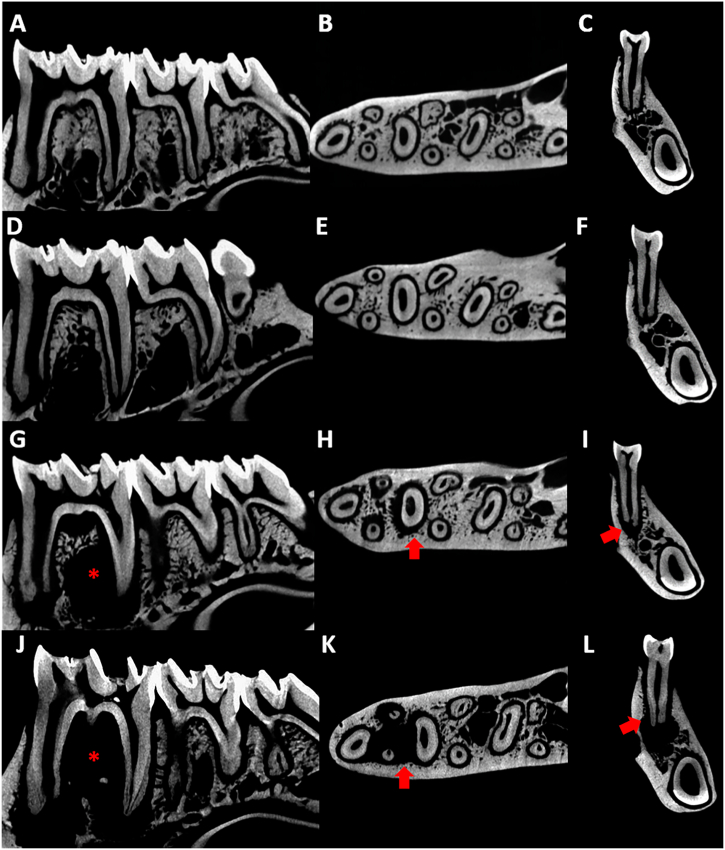
Representative microtomographs, being the control group from A-C, in sagittal (A), transverse (B) and coronal (C) sections; EtOH group from D-F, in sagittal (D), transverse (E) and coronal (F) sections; PA group from G-I, with sagittal (G), transverse (H), and coronal (I) sections; and PA + EtOH group from J-L, in sagittal (J), transverse (K), and coronal (L) sections. The arrows indicate the increased periodontal ligament space in the region of the root with the lesion, whereas the asterisks mark the volume of bone loss by the periapical lesion.

In a qualitative approach, it is already possible to observe the presence of alveolar bone alterations in different sections such as sagittal, transversal, and coronal. When looking at the microtomographic sections of the control group (Fig. 5 A, B, and C), and the group exposed to EtOH (Fig. 5 D, E, and F), the radiolucent area observed refers to the periodontal ligament space. However, when viewing the group with only the periapical lesion (Fig. 5 G, H, and I) it is already possible to see that there is a well-established apical lesion, indicated by the increase in the proportion of the radiolucent area. When we combine the two conditions, PA + EtOH (Fig. 5 J, K, and L), it is clear that the alcohol contributed to the progression of the lesion by accentuating the difference between the area of remaining alveolar bone.

**Fig. 6:**
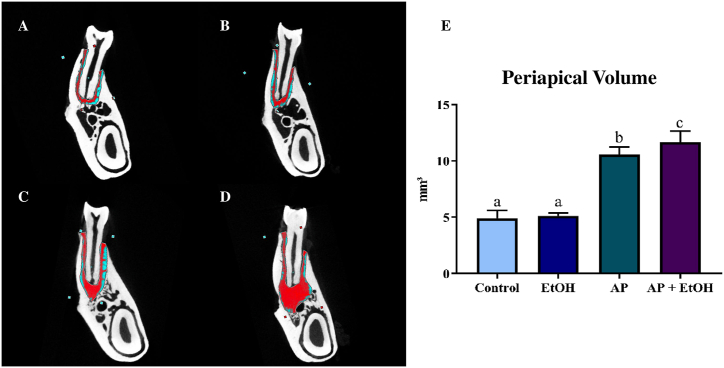
Result of microtomographic analysis using periapical volume as a parameter. From A-D representative MicroCT scans of the groups, control (A), Ethanol (EtOH) (B), apical periodontitis (C), and Apical Periodontitis + EtOH (D), the area delimited in red is measured for periapical volume assessment. In E, the graph of the statistical analysis using one-way analysis of variance (ANOVA), post-Tukey test (p < 0.05). In the graph, equal letters indicate no statistical difference, whereas different letters mean statistically significant differences.

A quantitative approach on measuring the volume of the lesion, the AP group (10.6 ± 0.239) showed a significant increase in the volume lesion when compared to the control (4.92 ± 0.30 mm^3^; p = 0.0001) and EtOH (5.11 ± 0.11 mm^3^; p = 0.0001) groups. In contrast, the high-intensity drinking EtOH administration *per se* did not increase the spaces inter trabecular with statistically similar to the control group (p = 0.9764). Interestingly, a statistically significant difference was observed between the groups (Fig. 6E), showing an increase in volume in the ligament space and bone loss around the roots in the AP + EtOH group (11.7 ± 0.39) compared to AP (10.6 ± 0.239; p = 0.0365), control (4.92 ± 0.30 mm^3^; p < 0.0001), and EtOH (5.11 ± 0.11 mm^3^; p < 0.0001) groups.

### 3.2 EtOH consumption in the high-intensity drinking pattern altered the bone quality of rats with induced periapical injury

#### 3.2.1 EtOH consumption in the high-intensity drinking pattern reduced the percentage bone volume of rats with induced periapical injury

**Fig. 7:**
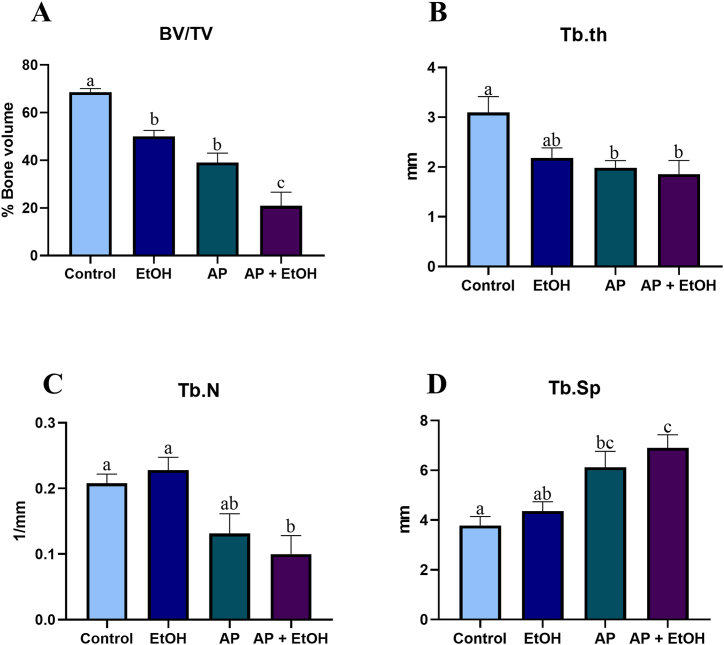
Results of microtomographic analyses of bone quality assessment, where A is the parameter of bone volume, B is trabecular thickness, C number of trabeculae per millimeter, and D is the spacing between trabeculae. Statistical analysis by one-way analysis of variance (ANOVA), after Tukey's test (p < 0.05). In the graphs, equal letters indicate no statistical difference, whereas different letters mean statistically significant differences.

In the analysis of the percentage of bone filling (bone volume/tissue volume), shown in Fig. 7A, to confirm our pattern of periapical lesion induction, we can see that there was a reduction in bone volume in the AP group (39.03 ± 3.9) when compared to the control group (68.57 ± 1.5; p = 0.0015). Interestingly, we noticed a reduction in % BV/TV when comparing the control group (68.57 ± 1.5) with the EtOH-exposed group (50.06 ± 2.5; p = 0.0482). When analyzing the PA + EtOH group (20.88 ± 5.7), it was possible to see a considerable reduction in bone volume compared to all the other groups: control (64.81 ± 2.7; p < 0.0001), exposed to EtOH (20.88 ± 5.7; p = 0.0006), and exposed to PA (39. 03 ± 3.9; p = 0.0297), indicating that the association between endodontic disease, when associated with high-intensity drinking consumption, is capable of accentuating the reduction in % bone volume.

#### 3.2.2 EtOH consumption in the high-intensity drinking pattern modulated the trabecular bone structure of rats with induced periapical injury

The analysis of Tb.Th, shown in Fig. 7B, resulted in a statistically significant reduction in the thickness of the trabeculae in the AP + EtOH group (1.85 ± 0.27 mm), when compared to the control group (3.09 ± 0.32 mm; p = 0.0091), similar to AP (1.98 ± 0.14 mm) compared to the control (p = 0.0211), whereas when AP + EtOH and AP groups were compared, no statistical differences were found (p = 0.9737). Nonetheless, no statistical difference was noted between the EtOH (2.18 ± 0.19 mm) and the control (p = 0.1187), AP (p = 0.9384), and AP + EtOH groups (p = 0.7798).

In the Tb.N analysis (Fig. 7C), the AP + EtOH group (0.10 1/mm ± 0.02) showed a significant reduction in the number of trabeculae compared to the control (0.20 1/mm ± 0.01; p = 0.0428) and EtOH (0.22 1/mm ± 0.01; p = 0.0139) groups, however, there was no statistical difference with the AP group (0.13 1/mm ± 0.02; p = 0.7973), nor was there between the control and EtOH groups (p = 0.9552).

As for the Tb. Sp, a statistically significant increase in trabecular spacing was observed in the AP + EtOH group (6.90 ± 0.52 mm) compared with the control (3.78 ± 0.36 mm; p = 0.0053) and EtOH (4.36 ± 0.37 mm; p = 0.0257) groups; this reduction was smaller in the AP group (6.12 ± 0.63 mm), showing a statistically significant difference only in comparison with the control group (p = 0.0296), as can be seen in Fig. 7D.

### 3.3 At 28 days following the injury induction, the EtOH animals exhibited more significant tissue damage

**Fig. 8:**
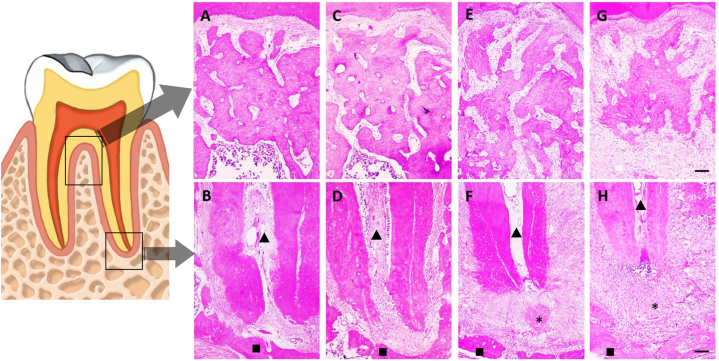
Representative photomicrographs of the alveolar bone in the furcation and apical regions. In A, furcation region; in B, apical region of the control group; in C, furcation region; and D, apical region of the EtOH group; in E, furcation region; and F, apical region of the PA group; in G, furcation region and H, apical region of the PA + EtOH group. The asterisks represent the area of the periapical lesion, the triangles indicate the root canal and the squares the alveolar bone. 40× magnification and 100 μm scale bar.

Comparing the experimental groups 28 days post-lesion induction revealed heightened tissue deterioration in animals treated with ethanol compared to those untreated, corroborating microtomography findings, as can be observed in Fig. 8. Micrographs also depicted greater root surface impairment in ethanol-treated animals versus those with periapical lesions but no ethanol exposure. Moreover, ethanol-exposed animals exhibited sparser collagen granulation tissue compared to their non-ethanol counterparts. Qualitatively, at the 28-day mark, there were no differences in the presence of osteoclasts between the two groups with apical periodontitis at the lesion site.

### 3.4 EtOH consumption in the high-intensity drinking pattern decreased the collagen content within the alveolar bone remaining of rats with induced periapical injury

**Fig. 9:**
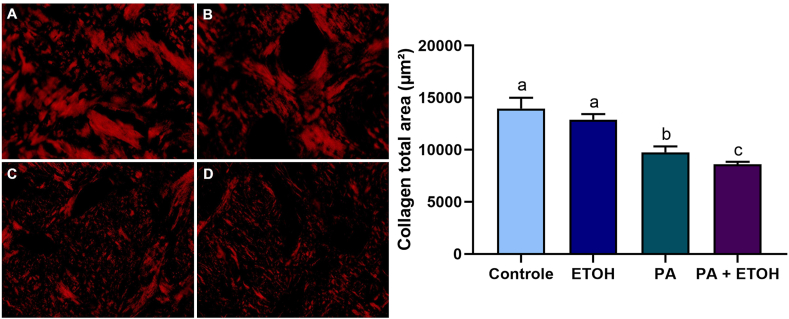
Demonstration of collagen preservation content through histochemical analysis with PicroSirius Red staining. Photomicrographs A, B, C, and D display representative images of the control, ethanol, apical periodontitis, and apical periodontitis with ethanol groups, respectively. The graph represents the total collagen area (μm^2^). Scale bar: 100 μm. The same letters indicate no significant differences between groups (p > 0.05). One-way ANOVA was employed, followed by the Tukey post hoc test for data analysis (n = 8).

Regarding the collagen content of the alveolar bone, the group with AP (9753 ± 573.3) showed a reduction compared to the control group (13963 ± 1036; p < 0.0001). The animals in the AP + EtOH (8595 ± 244) group showed a greater decrease in the amount of significant collagen in the remaining alveolar bone compared to the AP group (9753 ± 573.3; p = 0.0396). However, there was no significant difference in the EtOH group (12888 ± 537.4) compared to the control group (p = 0.1580), as shown in Fig. 9.

## 4 Discussion

Our results reject the null hypothesis and accept the alternative hypothesis, since they show that intermittent-excessive alcohol consumption during the progression of periapical disease aggravates bone destruction, promoting an increase in lesion volume and a decrease in bone volume and collagen content in the residual bone surrounding the lesion. It is well-recognized the relationship between alcohol exposure and periodontitis in experimental and clinical studies, emerging as a modifiable factor of risk [[43], [44], [45], [46], [47]]. However, to the best of our knowledge, this is the first study to show that 4-cycles of extreme drinking during adolescence period can also aggravate AP.

Chronic alcohol consumption research has already demonstrated the adverse effects of EtOH in soft [48] and mineralized tissues [25], as well as in endodontic diseases [30,49]; however, according to recent epidemiological research, this typical pattern of chronic continuous alcohol consumption consists of a few groups of drinkers, which the excessive binge-types drinking profile has been considerably increased since the COVID-19 pandemic [21,22].

Social factors that affect levels and patterns of alcohol consumption include cultural and social norms, level of economic development and alcohol policies. Specifically, high-intensity drinking is mostly associated with specific populations, such as young adults, who are disproportionately more negatively affected by alcohol abuse [18]. The importance of studying this topic transcends individual health concerns, as it deeply affects society as a whole. By examining this consumption pattern, we can better understand how public health interventions, educational programs, and regulatory policies can be designed to mitigate its negative impacts, promoting greater collective well-being and social equity [50]. Additionally, identifying specific social determinants allows these interventions to be more targeted and effective, especially among populations that are often more vulnerable to the harmful effects of high-intensity drinking.

In the central nervous system, prenatal exposure to heavy continuous alcohol administration at the dose of 2.25, 4.5, and 6.5 g/kg of 22.5 % EtOH for 33 or 42 days, it was observed more consistent harmful effects in the 6.5 g/kg exposure [51,52]. Scarce studies have explored the variables of alcohol exposure *vs.* oral health *vs.* period of exposure. In scarce studies of prolonged prenatal heavy continuous drinking exposure models, offspring showed mandible mineralization changes, specifically Ca and P concentrations, as well as alterations in the skeletal units of the mandible [53,54]. Our group has shown that 1-cycle of high-intensity drinking during pregnancy elicited oxidative damage and morphometric changes in the salivary glands of the offspring [55]. These findings suggest that prenatal exposure at different protocols interferes with offspring oral health.

In adolescence stage, studies have reported that heavy continuous drinking for 55 days (i.e., 6.5 g/kg) also elicited damage to the nervous system [56,57]. Notably, our group demonstrated that 4-cycles of intermittent high-intensity drinking (3 g/kg/day of 20 % w/v) was sufficient to impair central nervous system tissue, with long-term repercussions [58,59]. In the stomatognathic system, prolonged and continuous heavy drinking protocol resulted in histological and morphometric changes in salivary glands, particularly gland-specific [40,41]. In the adolescent intermittent high-intensity (4-cycles) paradigm, rats exhibited biochemical and morphological changes in several components of oral activity, such as saliva quality, salivary glands, and alveolar bone, even in healthy animals [34,36,41,57,60]. Interestingly, alveolar bone negative effects were found in long-term withdrawal in adult life [34,35], as seen in the central nervous system damage [58,59]. In the induced-periodontitis protocol, the 4-cycles of extreme drinking aggravated the periodontitis-induced quality of alveolar bone damage [36]. These data highlight that alcohol dose is a relevant factor in alcohol-related problems regardless of age.

As discussed above, the intermittent, high-intensity, and episodic alcohol intake in the alveolar bone of healthy adolescent rats has shown that this form of ingestion can promote damage to mineralized tissue through changes in bone microstructural parameters, such as reducing the percentage of the ratio of bone volume to tissue volume, decreasing the thickness of the trabeculae, and increasing the space between the trabeculae [34,35]. Similarly, our results also showed a negative modulation of the microstructural parameters of alveolar bone caused by high-intensity episodic alcohol protocol, with reduction in the ratio of bone volume *versus* tissue volume in the alcohol-exposed *per se* group. This was intensified in the high-intensity alcohol intake model in association with AP, where we observed an even greater reduction in the ratio of bone volume to tissue volume and an intensification of the loss of trabecular thickness and the number of trabeculae, as well as a greater increase in the trabecular space. These microstructural changes can lead to a significant reduction in the quality of bone tissue, which may be related to the greater intensity of issues owing to AP when combined with alcohol intake, as observed in this study [61].

Concerning its effects on the alveolar bone, a recent study found that rats with periodontal disease exposed to this same binge-type protocol (3 g/kg/day; 3 days on-4 days off) showed a reduction in bone quality and a worsening in vertical bone loss caused by the disease [36]. However, there is a need for further research on the association between high-intensity drinking and endodontic diseases, particularly periapical lesions. In this study, the lesion induction process followed the parameters already validated in the literature [39,62], which included access to the pulp chamber only, leaving it exposed to microorganisms. A previous study [63] demonstrated that whale teeth that suffer pulpal exposure owing to masticatory factors, even without direct injury to the pulp chamber, develop endodontic disease because of the exposure of the tissue to the external environment. Thus, the model exposes the pulp tissue to the oral cavity for 28 days in rats [64] ensuring that the process of pulp necrosis occurs gradually towards the apex, where inflammation begins in the third day after induction [65]. There is a decrease in odontoblasts and an increase in osteoclasts in the periradicular region; thus, initiating bone resorption and the acute phase of the disease [64,66], which lasts until the 21st day after induction in the rats.; the disease enters in the chronic phase until established after 28 days of induction [67].

The immune-inflammatory response in apical periodontitis (AP) is a dynamic and complex process. Initially, the innate and adaptive immune system is activated by bacterial biofilms present in root canals, which leads to the recruitment of immune cells to the periapical area and the release of large amounts of inflammatory mediators [68]. These pro-inflammatory mediators, such as cytokines (interleukins 1, 6, 11, 17 and 20) and tumor necrosis factor alpha (TNF-α), intensify inflammation by signaling bone resorption and inhibiting the formation of new bone tissue [[69], [70], [71], [72], [73], [74]]. In addition, oxidative stress and dysregulated metabolism also contribute to the destruction of periapical tissue. The persistence of these stimuli causes an over activation of the immune response, inducing the differentiation of osteoclasts, inhibiting the differentiation of osteoblasts and, consequently, leading to periapical bone resorption. The balance between pro- and anti-inflammatory responses in the periapical microenvironment is therefore the determining factor in the progression or regression of AP [68].

Additionally, concerning the development of the disease, some host intrinsic factors and habits known as disease modifiers, influence the lesions of AP [75] affecting the long-term persistence of the inflammatory disease at a chronic level [76,77]. Besides, alcoholism and smoking habits promote oral and immunological alterations, contributing to variations in the inflammatory process, increasing the severity response [78,79]. Regarding alcohol intake and periapical lesions, the literature shows that chronic consumption can exacerbate the inflammatory response and interfere with the RANK/RANKL system, proinflammatory cytokines, and mTOR signaling that play a fundamental role in bone metabolism, acting on osteoclastogenesis, whereas osteoprotegerin protects the bone against excessive resorption [[78], [79], [80]]. Upon alcohol exposure, the expression of RANKL is accentuated, favoring the differentiation of osteoclasts and inhibiting their apoptosis; thus, aggravating the process of bone resorption caused by endodontic diseases [30].

Therefore, when alcohol consumption coincides with pathogen exposure, EtOH hinders the elimination of toxic substances from the bloodstream, preventing the maintenance of metabolic homeostasis and altering the organism's defense mechanisms [81]. This is partly because alcohol inhibits the function of antigen-presenting cells and affects the differentiation of progenitor B cells by blocking transcription factors, which contributes to a reduced acquired immune response and increased susceptibility to infections [82,83]. Moreover, significant increases in neutrophils, basophils, monocytes, leukocytes, and polymorphonuclear cells may occur [30,84]. In addition, there is evidence in the literature to support the presence of an abnormally high profile of circulating cytokines during alcohol consumption [85]. Especially pro-inflammatory cytokines, which are part of the inflammatory process of endodontic disease, such as the peripheral cytokines IL-6 [86], IL-8, IL-10, IL-12 [87], as well as TNF-α [88], as well as C-reactive protein (CRP), have been implicated in alcoholics [89].

Furthermore, EtOH can modulate the redox system, promoting the formation of reactive oxygen species and reducing antioxidant activities, such as catalase and superoxide dismutase via. Such negative effects cause an oxidative imbalance, with free radicals overproduction, leading to oxidative stress and consequently, mitochondrial damage, cytotoxicity, and cell death, which is one of the main mechanisms of damage caused by alcohol intake [41,[90], [91], [92]]. Although chronic alcohol consumption has different periods of exposure, the mechanism of alcohol damage is similar but dose-dependent; that is, the intensity of the damage is directly related to the consumption pattern. Therefore, AP-related bone damage in association with high-intensity drinking consumption is similar to that occurring in chronic alcohol consumers and can aggravate the damage related to induced AP [16,30,32].

AP is associated with a bacterial infection within the root canal system, leading to collagen degradation, primarily by the action of matrix metalloproteinases. These enzymes play a pivotal role in collagen breakdown, which consists of the main protein subjected to degradation during the progression of periapical periodontitis [93]. To highlight the effect of experimental alcohol consumption on the organic matrix, systemic metabolic changes and bone turnover are well-documented in the literature, especially concerning microarchitecture and bone mineral quality [36,94]. However, the data indicated that EtOH *per se* did not induce changes in collagen in the extreme drinking pattern. In the occurrence of EtOH combined with AP, exacerbation of collagen damage was observed compared to AP alone.

Evidence suggests that, in the presence of chronic alcohol consumption, the organic matrix decreases bone density and exacerbates the inflammatory response and osteoclastogenesis in the periapical region; thus, favoring the development of AP [16,30,32]. Changes in bone quality result from multifactorial interactions. These modifications are mainly associated with changes in the mineral matrix, collagen, and non-collagenous proteins that regulate the arrangement of the bone matrix [94]. The combination of the inflammatory effects of AP and EtOH may have a synergistic effect, leading to greater collagen degradation and compromising bone tissue repair mechanisms. In fact, there is a lack in literature to compare the hazardous effects of ethanol exposure according to the protocol of exposure on the stomatognathic system.

This study has a few limitations. Our study shows that high-intensity drinking aggravates the progressing AP lesion; however, additional questions emerge. It is questionable whether the same findings would be found if the periapical disease was already established or what is the most harmful pattern of ethanol exposure. And from this perspective, whether the consumption of EtOH prior to endodontic disease would be able to influence the progression of the periapical lesion as found in this study. Similarly, our study failed to determine whether EtOH can modulate lesion progression even during periods of EtOH abstinence following successive binge-type cycles. Therefore, based on the results of this research, the raising of such questions and considering the importance of advancing the understanding of this field, it is essential that further studies are carried out employing methodologies in addition to those already mentioned. These studies should involve exploring the gene expression and molecular patterns of cytokines and other inflammatory molecules involved in the pathological mechanisms related to alcohol consumption. In order to better elucidate the possible association between EtOH in the form of high-intensity consumption and endodontic disease, as well as to verify the different possibilities of time and form (high-intensity or heavy drinking) of consumption in relation to the development of endodontic disease.

## 5 Conclusion

EtOH exposure, in a pattern of high-intensity drinking consumption, can aggravate AP, as evidenced by the increase in lesion volume and, consequently, bone loss. Additionally, EtOH consumption modified bone quality parameters, such as reduced bone volume, thickness, and number of trabeculae, as well as increased the spacing between bone trabeculae in AP-induced rats.

## CRediT authorship contribution statement

**José Mário Matos-Sousa:** Writing – original draft, Software, Methodology, Investigation, Formal analysis, Conceptualization. **Deiweson Souza-Monteiro:** Writing – original draft, Visualization, Validation, Software, Methodology, Investigation, Formal analysis, Conceptualization. **Vinicius Ruan Neves dos Santos:** Methodology, Investigation, Formal analysis. **Maria Karolina Martins Ferreira:** Visualization, Validation, Supervision, Formal analysis. **Deborah Ribeiro Frazão:** Methodology, Investigation, Formal analysis, Conceptualization. **Victória Santos Chemelo:** Software, Methodology, Investigation. **Leonardo de Oliveira Bittencourt:** Writing – review & editing, Validation, Supervision, Formal analysis, Data curation, Conceptualization. **João Daniel Mendonça de Moura:** Writing – review & editing, Visualization, Validation, Methodology, Investigation, Formal analysis, Conceptualization. **Cristiane do Socorro Ferraz Maia:** Writing – review & editing, Funding acquisition, Formal analysis, Conceptualization. **Fabrício Mezzomo Collares:** Writing – review & editing, Software, Methodology, Investigation, Funding acquisition. **Luanna de Melo Pereira Fernandes:** Writing – review & editing, Visualization, Validation, Formal analysis. **Rafael Rodrigues Lima:** Writing – review & editing, Visualization, Validation, Supervision, Software, Resources, Project administration, Methodology, Investigation, Funding acquisition, Formal analysis, Data curation, Conceptualization.

## Funding

DRF and VSC received Brazilian Agency for Support and Evaluation of Graduate Education (10.13039/501100002322CAPES) scholarships, and LOB received a scholarship from 10.13039/501100005288Fundação Amazônia de Amaro a Estudos e Pesquisas (FAPESPA); R.R.L. and C.S.F.M. are a researcher from 10.13039/501100003593Conselho Nacional de Desenvolvimento Científico e Tecnológico (CNPq) and received grants numbered 312275/2021–8 and 303196/2022–0, respectively). Also, this research was funded by PROCAD Amazônia—10.13039/501100002322CAPES (23038.005350/2018–78).

## Declaration of competing interest

The authors declare that they have no known competing financial interests or personal relationships that could have appeared to influence the work reported in this paper.
